# Comparative assessment of three different indices of multimorbidity for studies on health-related quality of life

**DOI:** 10.1186/1477-7525-3-74

**Published:** 2005-11-23

**Authors:** Martin Fortin, Catherine Hudon, Marie-France Dubois, José Almirall, Lise Lapointe, Hassan Soubhi

**Affiliations:** 1 Department of Family Medicine, Sherbrooke University, Sherbrooke, Que, Canada; 2 Centre de Santé et de Services Sociaux de Chicoutimi, Que, Canada; 3 Department of Community Health Sciences, Sherbrooke University, Sherbrooke, Que, Canada; 4 Research Center on Aging, Sherbrooke University Geriatric Institute, Sherbrooke, Que, Canada

## Abstract

**Background:**

Measures of multimorbidity are often applied to source data, populations or outcomes outside the scope of their original developmental work. As the development of a multimorbidity measure is influenced by the population and outcome used, these influences should be taken into account when selecting a multimorbidity index. The aim of this study was to compare the strength of the association of health-related quality of life (HRQOL) with three multimorbidity indices: the Cumulative Illness Rating Scale (CIRS), the Charlson index (Charlson) and the Functional Comorbidity Index (FCI). The first two indices were not developed in light of HRQOL.

**Methods:**

We used data on chronic diseases and on the SF-36 questionnaire assessing HRQOL of 238 adult primary care patients who participated in a previous study. We extracted all the diagnoses for every patient from chart review to score the CIRS, the FCI and the Charlson. Data for potential confounders (age, sex, self-perceived economic status and self-perceived social support) were also collected. We calculated the Pearson correlation coefficients (r) of the SF-36 scores with the three measures of multimorbidity, as well as the coefficient of determination, R^2^, while controlling for confounders.

**Results:**

The r values for the CIRS (range: -0.55 to -0.18) were always higher than those for the FCI (-0.47 to -0.10) and Charlson (-0.31 to -0.04) indices. The CIRS explained the highest percent of variation in all scores of the SF-36, except for the Mental Component Summary Score where the variation was not significant. Variations explained by the FCI were significant in all scores of SF-36 measuring physical health and in two scales evaluating mental health. Variations explained by the Charlson were significant in only three scores measuring physical health.

**Conclusion:**

The CIRS is a better choice as a measure of multimorbidity than the FCI and the Charlson when HRQOL is the outcome of interest. However, the FCI may provide a good option to evaluate the physical aspect of HRQOL for the ease in its administration and scoring. The Charlson index may not be recommended as a measure of multimorbidity in studies related to either physical or mental aspects of HRQOL.

## Background

The coexistence of multiple chronic diseases in the same individual or multimorbidity has led to increasing interest in its measure in research studies as a potential confounder or as a predictor of study outcome [1,2].

Health-related quality of life (HRQOL) is an outcome measure that is adversely affected by the presence of multimorbidity. This association can be demonstrated using the simple count of chronic conditions as a measure of multimorbidity [3-8]. However, we found in a recent study that the use of a multimorbidity index, the Cumulative Illness Rating Scale (CIRS), revealed a stronger association of HRQOL with multimorbidity than a simple count of chronic diseases [8]. Measures of multimorbidity are often applied to source data, populations or outcomes outside the scope of the original developmental work [9]. However, as the development of a multimorbidity measure is influenced by the population and outcome used, these influences should be taken into account when selecting a multimorbidity index [10]. Although the CIRS is a comprehensive evaluation of medical problems by organ system, it was not developed in light of HRQOL. Therefore, it can be argued that another measure of multimorbidity (or comorbidity if an index disease is the object of study) specifically designed for HRQOL could bear a stronger relationship with HRQOL than the CIRS, and would be a better measure of multimorbidity when the outcome of interest is HRQOL.

Several indices have been described to measure multimorbidity or comorbidity [1,2,11]. However, some problems related to many of these indices have been reported such as insufficient data on their clinimetric properties and moderate inter-rater reliability [2,12]. Two indices stand out as potential alternatives to the CIRS, the Charlson Index and the Functional Comorbidity Index (FCI). The Charlson index [13] is, with the CIRS [14], among the most valid and reliable measures of multimorbidity [2]. The Charlson index is the most extensively studied comorbidity index and, although the weights originally used to develop it were based on the relative risk of dying, it has been found to significantly predict the number of ambulatory visits, the probability of an inpatient admission, the length of stay, and hospital costs [9,15]. However, the association between the Charlson index and HRQOL has been assessed only in patients of age 65 or older [16]. Recently developed, the Functional Comorbidity Index (FCI) [11] was specifically developed with physical functioning, an aspect of HRQOL, as the validity criterion. The index was developed using two databases totalizing 37,772 Canadian and US adults seeking treatment for spine ailments. It is possible that the association of this index with physical aspects of HRQOL could outperform the CIRS, but this hypothesis has not been tested yet.

Using these three indices (CIRS, FCI and Charlson) on the same target population would allow a better comparison of their performance when the outcome of interest is HRQOL, but we could not find any study with such comparison. Thus, the primary purpose of this study was to compare the strength of the association of the CIRS, the Charlson index and the FCI measures of multimorbidity, with HRQOL.

## Methods

We used data collected on the diagnoses of chronic diseases in a group of 238 adult primary care patients (age 18 or older) who participated in a study on HRQOL [8]. Patients were recruited from the clientele of 21 family physicians in the Saguenay region, Canada. Details of the sampling are described elsewhere [17]. In brief, we randomly selected patients from 980 patients who had also been selected at random for a prevalence study on multimorbidity [17]. Our goal was to recruit 60 patients for each CIRS quintile to have enough representation of different levels of multimorbidity. Of the 419 patients we tried to contact by phone, 66 could not be reached, despite repeated attempts. Of the remaining 353 patients, 238 agreed to participate (Table 1). Patients completed the self-administered 36-item short form of the Medical Outcomes Study questionnaire (SF-36) [18] to assess HRQOL. The SF-36 comprises 8 multi-item scales divided into 2 main groups: physical and mental aspects of quality of life. Two summary scores for each group are obtained through a weighted sum of these scales. To compute the Physical Component Summary scale, high positive weights are given to the scales of the physical aspects of quality of life and low negative weights to those of the mental health. To calculate the Mental Component Summary scale, low negative weights are given to the scores of the physical aspects of quality of life and high positive weights are given to those of the mental health. For all scales and both summary scales, lower scores indicate lower HRQOL.

**Table 1:** Characteristics of the Sample

**Characteristic**	**Refusals (n = 115)**	**Participants (n = 238)**	***P *value**
Mean (SD) age, y	56.5 (17.4)	59.0 (14.3)	0.17*
Mean (SD) diagnoses, n	5.5 (3.2)	5.3 (2.8)	0.49*
Male, %	33.9	29.0	0.39^†^

*t-test.

^†^Chi-square test.

From an exhaustive chart review, we extracted a comprehensive list of diagnoses of all chronic conditions for every patient after informed consent. We then used the list to score the CIRS [19], the FCI [11] and the Charlson index [13] (Table 2). To obtain the most reliable measures for analysis, the three indices were scored by two investigators independently in a group of patients (the number of patients varied from 49 to 73 for the 3 indices), and inter-rater reliability was calculated. During a standardization period, the scoring process was discussed to reach a consensus and repeated until the inter-rater reliability was judged acceptable [20].

**Table 2:** Main characteristics of CIRS, FCI and Charlson†

**Comorbidity indices**	**CIRS**	**FCI**	**Charlson**
**Items**	1. Cardiac 2. Vascular 3. Hematological 4. Respiratory 5. Ophthalmological and ORL 6. Upper gastrointestinal 7. Lower gastrointestinal 8. Hepatic and pancreatic 9. Renal 10. Genitourinary 11. Musculoskeletal and tegumental 12. Neurological 13. Endocrine, metabolic, breast 14. Psychiatric	1. Arthritis (rheumatoid and osteoarthritis) 2. Osteoporosis 3. Asthma 4. COPD, ARDS* 5. Angina 6. Congestive heart failure or heart disease 7. Heart attack 8. Neurological disease 9. Stroke or transient ischemic attack 10. Diabetes types I and II 11. Peripheral vascular disease 12. Upper gastrointestinal disease 13. Depression 14. Anxiety or panic disorders 15. Visual impairment 16. Hearing impairment 17. Degenerative disk disease 18. Obesity and/or BMI > 30 kg/m^2^	1. Myocardial infarct 2. Congestive heart failure 3. Peripheral vascular disease 4. Cerebrovascular disease 5. Dementia 6. Chronic pulmonary disease 7. Connective tissue disease 8. Ulcer disease 9. Stroke or transient ischemic attack 10. Diabetes 11. Hemiplegia 12. Moderate or severe renal disease 13. Diabetes with end organ damage 14. Any tumor 15. Leukemia 16. Lymphoma 17. Moderate or severe liver disease 18. Metastatic solid tumor 19. AIDS

**Weights**	All systems weighted from 0 to 4: 0 No problem 1 Mild 2 Moderate 3 Severe 4 Extremely severe	Presence (yes) or absence (no) of diagnoses	Conditions from 1 to 10, weight = 1 Conditions from 11 to 16, weight = 2 Condition 17, weight = 3 Conditions 18 and 19, weight = 6

**Final score**	Sum of weights assigned to each system	Sum of "yes" answers	Sum of weights assigned to each condition that a patient has

† CIRS = Cumulative Illness Rating Scale; FCI = Functional Comorbidity Index; Charlson = Charlson index.

* COPD = chronic obstructive pulmonary disease; ARDS = acquired respiratory distress syndrome

Data for potential confounders (age, sex, self-perceived economic status and self-perceived social support) were also collected. Self-perceived social support was measured with the Social Provisions Scale [21]. The research ethics board of the Centre de santé et de services sociaux de Chicoutimi approved this study.

### Statistical analysis

To investigate the relationship between HRQOL and the multimorbidity indices as well as the direction of the relationships (positive or negative), we first calculated the Pearson correlation coefficients of the SF-36 scores with the three measures of multimorbidity. We also compared CIRS correlation coefficients with those of the FCI and the Charlson index [22]. Next, the coefficient of determination, R^2^, was calculated to measure the percentage of variation in the dependent variables (all SF-36 scales and two SF-36 summary scores) explained by each measure of multimorbidity over and above that explained by age, gender, self-perceived social support and self-perceived economical status. We obtained these estimates through multiple regression analysis for which underlying assumptions were judged satisfactory. All analyses were done using the SAS system for Windows (version 8.02, SAS Institute, Inc, Cary, NC, USA).

## Results

After standardization of the scoring process, the intraclass correlation coefficients for the inter-rater reliability were 0.96, 0.92 and 0.90 for the CIRS, the FCI and the Charlson respectively.

**Figure 1:**
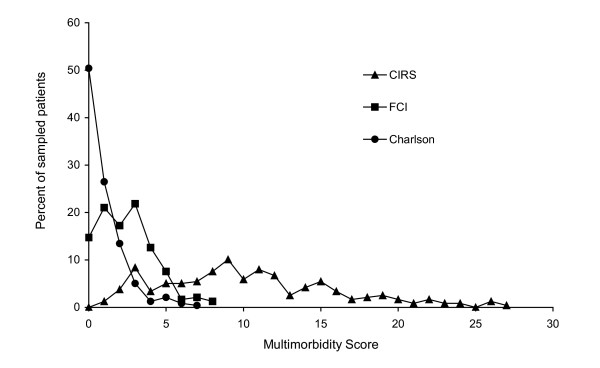
Distribution of scores on multimorbidity measures. CIRS = Cumulative Illness Rating Scale; FCI = Functional Comorbidity Index; Charlson = Charlson index.

Figure 1 shows the distribution of each multimorbidity score. The CIRS had the widest variation, with a range of 1 to 27 with a mode of 9 (mean = 10.3). The FCI had a range of 0 to 8, with a mode of 3 (mean = 2.4). The Charlson index had a similar range (0–7) but a different distribution from that of the FCI, with 120 patients (50.4%) having a score of zero (mean = 0.9).

Pearson correlation coefficients of SF-36 with the three measures of multimorbidity are shown in Table 3. The CIRS was negatively correlated with all scales of SF-36 except the Mental Component Summary; i.e. higher morbidity or multimorbidity level was associated with lower HRQOL. The FCI was negatively correlated with all SF-36 scales measuring the physical aspect of HRQOL; it was also negatively correlated with two scales measuring the mental aspect of HRQOL. The Charlson index was negatively correlated with all scales of SF-36 evaluating the physical aspect of HRQOL; it was not correlated with any of the scales evaluating the mental aspect. There was an unexpected positive correlation of the Charlson index with the Mental Component Summary that did not have any meaningful interpretation. The CIRS correlation coefficients were significantly different from those of the FCI for the SF-36 scales of Physical Functioning, Role Physical and Social Functioning as well as for the Physical Component Summary; whereas the Charlson correlation coefficients were significantly different from those of CIRS for all SF-36 scales.

**Table 3:** Pearson correlation coefficients of the SF-36^† ^scores with the measures of multimorbidity

**HRQOL (SF-36)**	**Correlation coefficients (r)**		
**Physical Health**	**CIRS‡**	**FCI**	**Charlson**
Physical Functioning	-0.55**	-0.47**	-0.31**
Role physical	-0.41**	-0.32**	-0.14*
Bodily Pain	-0.38**	-0.33**	-0.16*
General Health	-0.40**	-0.34**	-0.21**
**Mental Health**			
Vitality	-0.30**	-0.23**	-0.08
Social Functioning	-0.29**	-0.21**	-0.04
Role Emotional	-0.18**	-0.10	+0.03
Mental Health	-0.18**	-0.14**	+0.07

**Physical Component Summary**	-0.54**	-0.47**	-0.31**
**Mental Component Summary**	-0.06	-0.001	+0.16*

^† ^SF-36 = Self-administered 36-item short form of the Medical Outcomes Study questionnaire.

‡ CIRS = Cumulative Illness Rating Scale; FCI = Functional Comorbidity Index; Charlson = Charlson index. * p < 0.05; ** p < 0.01

Table 4 shows the percentage of variation in HRQOL explained by each measure of multimorbidity over and above that explained by age, gender, self-perceived social support and economic status. The CIRS explained the highest percent of variation in all scores, except for the Mental Component Summary score where the explained variation was not significant.

**Table 4:** Percentage of variation of the SF-36 ◇ scores explained by each measure of multimorbidity

**HRQOL (SF-36)**	**Percentage of variation explained by the control variables†**	**Partial R^2^(%)‡**		
**Physical Health**		CIRS§	FCI	Charlson
Physical Functioning	21.08**	15.59**	9.53**	4.52**
Role physical	7.84**	11.14**	5.21**	0.56
Bodily Pain	10.02**	9.91**	6.80**	1.04
General Health	11.63**	14.07**	7.96**	2.99**
**Mental Health**				
Vitality	10.68**	6.78**	2.56*	0.10
Social Functioning	10.77**	6.72**	2.94**	0.002
Role Emotional	6.71**	2.64*	0.43	0.52
Mental Health	17.07***	2.26*	1.02	1.18

**Physical Component Summary**	13.18**	17.75**	11.81**	5.46**
**Mental Component Summary**	12.60**	0.75	0.02	2.80

◇ SF-36 = Self-administered 36-item short form of the Medical Outcomes Study questionnaire.

† control variables: age, gender, self-perceived social support and self-perceived economical status

‡ controlling for age, gender, self-perceived social support and self-perceived economical status.

§CIRS = Cumulative Illness Rating Scale; FCI = Functional Comorbidity Index; Charlson = Charlson index.

* p < 0.05 ; ** p < 0.01

## Discussion

We compared the strength of association of three multimorbidity indices (CIRS, FCI and Charlson index) with HRQOL as the outcome of interest in a primary care context. In terms of percent of explained variation in HRQOL, the CIRS performed as well as and often better than the FCI and the Charlson index in all scores of the SF-36. Correlation coefficients of the SF-36 scores with the measures of multimorbidity were always higher for the CIRS, followed by the FCI (Table 3); the correlations of the SF-36 scores with the Charlson index were always the weakest. We also found an unexpected positive correlation of the Charlson index with the SF-36 Mental Component Summary.

Among the three indices, the CIRS was the one that explained the highest percent of variation in all scores of the SF-36. Despite the fact that the FCI was developed with physical function as the outcome of interest, it did not perform better than the CIRS in any of the scales of the SF-36 evaluating the physical aspect of HRQOL. This result may be due in part to the wider range of possible scores on the CIRS. Indeed, an index ranging from 0 to 27 can better predict variations in an outcome than one that ranges from 0 to 7 or 8 with more than half the patients being classified in the first 2 or 3 levels of the scale. It may also be due to the fact that the CIRS evaluates the number and severity of all chronic diseases whereas the FCI evaluates a limited number of diagnoses and does not take into account disease severity. However, R^2 ^values for the FCI related to physical health scores, although lower than those of the CIRS, remained highly significant after controlling for confounders. Given that the FCI is very easy to administer and score, researchers may consider, depending on the characteristics of the study, to trade off a lower explained variation for simplicity to evaluate the physical aspect of HRQOL. In the case of the Charlson index, the percent of explained variation was significant only in the Physical Functioning, the General Health, and the Physical Component Summary scales. In the mental aspect of HRQOL, the percent of variation explained by the Charlson index was not significant in any of the scales of the SF-36. Given these results, the Charlson index may not be recommended as a measure of multimorbidity in HRQOL studies in adults.

The FCI was the only index of multimorbidity that we were aware of that was developed using a component of HRQOL (Physical Functioning) as outcome. However, two other articles reporting multimorbidity measures related with HRQOL were published upon completion of the present study. One of the articles describes a new self-reported assessment of comorbidity, or self-reported disease burden [16]; the other article describes five indices or approaches to scoring multimorbidity derived from a self-administered multimorbidity questionnaire [23].

In the article on the self-reported disease burden [16], the index was validated using two scales of the SF-36 evaluating the physical aspect of HRQOL (Physical Functioning and one item of General Health) as well as the outcomes of depression and self-efficacy. The authors studied these outcomes using the Charlson index and the findings were similar to ours. They found a negative correlation between the Charlson index and the Physical Functioning and General Health outcomes [16]. However, our study expanded the analysis of the Charlson index to all scales of the SF-36 evaluating both physical and mental aspects of the HRQOL. Moreover, we included adults aged 18 and over, whereas age was restricted to 65 years or older in the study on the self-reported disease burden [16]. In the second paper by Byles et al [23], the study was a comparison of the performance of five indices derived from a self-administered multimorbidity questionnaire. None of the indices was compared to other indices previously published. Unfortunately, it was not possible to include these five indices in our comparative study because of the chart review method that we used. However, future research comparing CIRS with these five indices as well as with the self-reported disease burden index is warranted.

In our analysis of the relationship between mental aspects of HRQOL and multimorbidity, we found some contradictory results that may reflect a limitation in our instruments. All scales of the SF-36 used to measure the mental aspect of HRQOL were related to the CIRS, whereas the Mental Component Summary was not (Tables 3 and 4). This summary score was created by the developers of the SF-36 with the hope to reduce the number of statistical comparisons involved in analyzing the SF-36 without substantial loss of information [24]. The lowest possible score of the Mental Component Summary indicates frequent psychological distress, social disability due to emotional problems, and a poorly self-rated health [24]. However, the lack of relationship we found between the CIRS and the Mental Component Summary contradicts the relationship we found between the CIRS and all mental scales of the SF-36 of which the Mental Component Summary is a composite. One possible explanation may be that the calculation of the Mental Component Summary takes into account not only the four scales measuring mental health, but also the four scales measuring physical health which are weighted negatively [25]. As a result, the positive weights of the mental health scales may be canceled out by the negative weights of the physical health scales which have a stronger relationship with the CIRS in our study. This problem was evident in the relationship between the CIRS and the Mental Component Summary, but it also affected the relationships between this summary score and the other measures of multimorbidity. These results suggest that the Mental Component Summary produced a substantial loss of information in the context of our study.

## Conclusion

In summary, our study suggests that the CIRS is a better choice as a measure of multimorbidity than the FCI and the Charlson index in a primary care context when HRQOL is the outcome of interest. However, if researchers were interested only in the physical aspect of HRQOL, then the FCI, despite its lower explained variation in HRQOL, may provide a good option for the ease in its administration and scoring. Finally, based on our results, the Charlson index may not be recommended as a measure of multimorbidity in studies related to either physical or mental aspects of HRQOL.

## Authors' contributions

MF participated in the conception and design of the study, supervised data collection and analysis and drafted the manuscript. CH participated in the conception and design of the study and data analysis and helped draft the manuscript. M-FD participated in the design of the study, performed the statistical analysis and helped draft the manuscript. JA participated in the data analysis and helped draft the manuscript. LL participated in the data analysis and helped draft the manuscript. HS participated in data analysis and critically reviewed the manuscript. All authors gave their final approval of the version of the manuscript submitted for publication.
